# Chemical residue trends for Australian and New Zealand wool

**DOI:** 10.1038/s41598-022-04787-x

**Published:** 2022-01-14

**Authors:** Stephen Ranford, Paul Swan, Chikako van Koten

**Affiliations:** 1 AgResearch Limited, Private Bag 4749, Christchurch, 8140 New Zealand; 2 Paul G. Swan and Associates Pty Ltd, Sydney, Australia

**Keywords:** Applied mathematics, Statistics, Biochemistry, Health care, Chemistry

## Abstract

Textile consumer trends towards improved product safety and high environmental standards have significantly influenced regulators in key consumer markets. The apparel wool industry sector has responded to regulators, and for three decades the Australia and New Zealand wool industries have managed advancements in ectoparasiticides and improved sheep treatments targeting high environmental, animal health and welfare standards leading to safe wool products. Australian and New Zealand chemical residue data from greasy wool have been consolidated and analysed for organophosphate, synthetic pyrethroid, insect growth regulator, neonicotinoid, macrocyclic lactone and spinosad active. Trend analysis has been applied to time domain data to evaluate advancements in ectoparasiticide technology after revising environmental, animal health and welfare standards. Analysis shows impacts from technology improvement, regulatory change and compliance by sheep farmers meeting or exceeding published European Union residue limits for regulated ectoparasiticides namely organochlorine, organophosphate, synthetic pyrethroid and insect growth regulators. Implications from advancements in ectoparasiticide technology, industry management and regulatory measures, include healthy sheep growing in clean pastoral environments with evidence of reduced wool residue levels which complement high and rising proportions of Australian and New Zealand wool fibre meeting European Union Ecolabel criteria.

## Introduction

Wool production has been a historically and culturally important contributor to the world market leaders such as Australia and New Zealand for around 200 years^1^, and also to the global wool textile industry. New Zealand produced an estimated 139 × 10^6^ kg of greasy wool in 2018–2019, from 27.5 million sheep of which approximately 4.5% were Merino^2^. By comparison in the same period Australia generated 300 × 10^6^ kg of greasy wool from 72.5 m sheep, of which approximately 85% were Merino or allied breed derived^3^.

Sheep farmers in both countries confront a wide range of animal health challenges, and typically apply veterinary chemicals for preventative health reasons^4,5^ and for remedial treatment of acute conditions, such as in the event of an injury. In the case of ectoparasiticide chemicals, which are used to treat external parasites, proprietary formulations are developed to generate both high levels of treatment efficacy for well-defined periods, and also known breakdown profiles for the active compounds. The latter underpin the mandatory withholding period definitions in product labels, as part of the regulatory approval schemes implemented in Australia^6^ and New Zealand^7^. The chemical treatments for managing ectoparasiticides usually contain carbon, hydrogen, oxygen and some contain sulphur, while a few contain fluorine, chlorine, iodine and phosphorous. These chemicals are often molecular ring structures and the simpler generic name is reflected in the chemical name. For example, cyromazine(CYR), an insect growth regulator, is a triazine ring structured chemical^8^.

These chemicals pose a challenge for the industry, in the context of human health (e.g. managing exposure of farm workers and wool handlers to residual chemicals on greasy wool), but also in early stage wool processing, where the wool grease containing these chemical actives is separated from clean wool. Over the past decades, regulators have applied increasingly stringent environmental discharge standards to wool processors in Asia^9–14^, North America^15,16^ and within and across the European Union (EU)^17,18^. In addition to these point of discharge regulations, consumer product eco-labels are now providing an additional compliance requirement for greasy wool producers.

The EU Ecolabel, or EU Flower, is a voluntary ecolabel scheme established in 1992 by the European Commission. It was implemented in 2009^19^ as a voluntary ecolabel award scheme intended to promote products with a reduced environmental impact during their entire life cycle, and to provide consumers with accurate, non-deceptive, science-based information on the environmental impact of products based on peer reviewed scientific principles.

Life cycle assessment (LCA) science has been developed comprehensively since 1992^20^ and recently work has advanced by incorporating chemical toxicity impact categories into LCA assessments. Prior to 1992 the wool industries in both Australia and New Zealand had already implemented chemical residues surveys to assess the impact of new procedures and products with the aim of managing chemical residues. These initiatives have undoubtedly improved knowledge of chemical residue management.

With increasing concerns for the environment, monitoring is important for wool producers aiming to meet published pesticide residue limits. Legislation has lowered the acceptable chemical residues on wool, and some ectoparasiticides have been banned altogether. Because of this, the International Wool Textile Organisation (IWTO) saw a necessity to develop a method to test for chemical residues on greasy wool. Currently, a draft test method, IWTO DTM-59: 2009^21^, outlines tests for the presence of four classes of chemical residues: organochlorine(OC), organophosphate(OP), synthetic pyrethroid(SP) and insect-growth regulator(IGR). Several industry surveys were carried out in Australia and New Zealand to establish systematic approaches to sampling, measuring, monitoring and reporting residue levels to the wool trade, processors, industry regulators and government authorities.

Factors influencing lower residue trends include, (i) training and education relevant to pesticide use, (ii) observance of withholding periods between active applications and shearing designed to enable breakdown of residues, (iii) compliance with recommended application rates and, (iv) managing registration of pesticide actives used in the agriculture sectors. Measured OC residues in the surveys confirm deregistration of all organochlorine sheep products 27 and 22 years ago (Australia and New Zealand), has culminated in no survey result greater than the limit of reporting over the last 18 years.

While this analysis of residue surveys focuses on greasy wool shorn from sheep it is important to note that wool cleaning technology has advanced and consequently residues in commercially scoured wool fibre are significantly lower than the EU greasy wool limits discussed here^22^.

The EU Ecolabel scheme is part of the sustainable consumption and production policy of the European Community, which aims at reducing the negative impact of consumption and production on the environment, health, climate and natural resources. The scheme is intended to promote those products which have a high level of environmental performance through the use of the EU Ecolabel to limit the most significant environmental impacts of products during their whole life cycle. However, reflecting the constant evolution of veterinary pharmacology, a number of newer chemical classes in wide commercial use are not included in the EU-Ecolabel, such as CYR, imidacloprid (a neonicotinoid) (NEO), ivermectin (a macrocyclic lactone insecticide) (MLI)) and spinosad(SPI) ectoparasiticides^23^.

The regulatory framework applying to veterinary chemicals used for parasite control in the Australian and New Zealand wool industries involves the Australian Government Pesticides and Veterinary Medicines Authority (APVMA)^24^ and the New Zealand Ministry of Primary Industries Agricultural Compounds and Veterinary Medicines (ACVM)^25^, as the government statutory bodies responsible for the management of approvals for agricultural and veterinary chemical products in the two countries. The approval process examines human, animal and environmental risks of the product covering physico-chemical properties, toxicology, phytotoxicity, hazard assessment, labelling and withholding periods^26^ required between animal treatment and shearing.

In support of the Australian and NZ regulatory processes, and as a means of monitoring compliance with product use labels, surveys of ectoparasiticide residue levels on greasy wool have been underway more-or-less continuously in Australia since 1992, and periodically in New Zealand since 1987, using internationally accepted testing and proficiency systems established over the past 30 years for determining the levels of chemical residues in wool based on scientific method^21,27–38^. The earlier surveys were followed with an Australian initiative to monitor the newer chemical actives such as IGR and more recently the NEO and SPI actives. Australian data have been collected in National Wool Residues Surveys (NWRS) since 1992 (with an exception of no survey conducted in 2009)^39^. New Zealand data are available in industry reports dated back to 1988 and since 2015 the National Council of New Zealand Wool Interests participated in the New Zealand Wool Residue Survey over 2 seasons^40^ to establish contemporary data which are available to both the New Zealand and Australian wool industries.

However, the aggregated results of these surveys have not been previously published, instead being used for government and industry approval processes and to introduce new handling practices and extend education programmes aimed at reducing and eliminating both environmental, human and animal toxicity impacts.

Nonetheless, the lack of published data has not prevented organisations such as MADE-BY^41^ from generating assessments of human and eco-toxicity levels from greasy apparel wool. Such tools are intended to shape brand, retailer and ultimately consumer fibre choices to the extent that the data underpinning their ratings is not representative, not contemporary, and not subject to peer review.

Accordingly, the purpose of this paper is to provide accessible data summarising the status for the Australian and New Zealand wool, by aggregating previously unpublished historical residue data with contemporary results to enable analysis of trends. These trends encompass industry initiatives for managing ectoparasiticides and provide updated information on wool from these countries complying with EU residue limits.

## Methods

Wool harvested by shearers on farms was sampled from bales and tested for ectoparasiticide residues in each country by accredited and internationally licensed wool testing laboratories.

### Survey samples

The surveys utilised in this study were conducted during the 1988 to 2019 period and involved sampling of greasy wool bales pre-sale and during an annual wool growing and selling season using internationally accepted pre-sale wool bale coring regulations^37^. Wool types represented include fleece wool types from adult sheep, lamb’s wool and recently oddment types, such as bellies and pieces.

In Australia, industry surveys commenced in 1992 and from 2000 involved representative sampling of each wool type carried out in the 6 wool producing states. Each typically involved randomised collection and testing by the Australian Wool Testing Authority (AWTA) of between 300 and 600 samples (each sample taken represents a farm lot), stratified by month to adjust for the characteristic pattern of monthly wool auction offering in each state^39^.

The New Zealand data set consists of two early wool industry surveys completed in 1988 (80 samples) and 1993 (84 samples), and a major survey of farm lots randomly collected in each of the 2015/2016 and 2016/2017 wool production seasons by New Zealand Wool Testing Authority (NZWTA). For this survey, as in the Australian surveys since 2000, sample collection was stratified by calendar month within each of the main growing regions to provide a representative selection of samples from across the country^40^.

### Chemical testing

The samples were blended and analysed in accordance with the International Wool Textile Organisation (IWTO) Draft Test Method DTM-59 to determine chemical residues on greasy wool^21^. For this method, the limits of reporting (LoR) for each active are summarised in Table 1.

**Table 1:** Limits of reporting for residues analysis.

Active	LoR (ppm*)
Total organochlorine (OC)	0.05
Total synthetic pyrethroid (SP)	0.1
Total organophosphate (OP)	0.1
Sum (triflumuron + diflubenzuron + dicyclanil) (IGR_3_)	1.0
Cyromazine (CYR) or Spinosad (SPI)	1.0

*ppm = mg/kg.

From 2000, Australian tests were conducted at a single laboratory (AWTA Limited, Melbourne, Australia), and to minimise risk and impact of interlaboratory differences, the New Zealand samples collected in 2015/16 and 2016/17 were also analysed at this same facility.

### Statistical analysis

Frequencies at each concentration of the actives categories OP, SP, IGR, CYR, NEO, MLI, SPI covered in the 2016–2017 survey were analysed to determine the form of the residue distributions for recent wool data. The adjusted Fisher-Pearson coefficient of skewness (Eq. 1) and excess kurtosis (Eq. 2) where $\overline{Y }$ is the mean, *s* is the standard deviation , and *N* is the number of data points, were calculated for the distributions along with mean and median estimates, to provide evidence of the degree of asymmetry of the distributions (skew), and the proportion of outliers.

1 $$(\sqrt{N\left(N-1\right)})/(N-2)\times \left (\sum \limits_{i=1}^{N}{({Y}_{i}- \stackrel{-}{Y)}}^{3}/N)/{s}^{3} \right)$$

2 $$\left(\left(\sum \limits_{i=1}^{N}{({Y}_{i}-\stackrel{-}{Y)}}^{4}/N\right)/{s}^{4}\right)-3$$

The median is regarded as a robust statistic in a situation where there are substantially skewed distributions^42,43^, such as large pesticide databases^44^.

The mean and median residue concentration results were analysed each using locally weighted scatterplot smoothing regression (Lowess)^45,46^ by fitting polynomials to data with weighted least squares estimation, firstly for Australian data and thereafter with the addition of the more limited New Zealand data. The Lowess method provides smooth trend lines of mean and median residue levels of toxic chemical compounds, and the 95% confidence limits of the trends is used to indicate the level of uncertainty associated with the mean trend lines and residue predictions.

The Lowess model uses a goodness of fit summary statistic (Pseudo R^2^, Eq. 3) similarly to R^2^ in the simple linear regression approach. The residual standard error (RSE) is also calculated, which is the square root of mean residual sum of squares used to determine uncertainty of predictions.

3 $$\text{Pseudo} \; \text{R}^{2} \; ({\%}) = \left(1-\left(\sum \limits_{i=1}^{n}residuals/{\sum }_{i-1}^{n}{\left({y}_{i}-\overline{y }\right)}^{2}\right) \right)*100$$

Similarly, smooth trend lines of percentages of surveyed samples with their mean residue concentrations lower than the EU-Ecolabel limits were obtained over the time domain with Lowess, together with the 95% confidence limits and goodness of fit summary statistics.

## Results

Table 2 lists the pesticide concentration limits for individual active compounds implemented by the EU-Ecolabel system, along with actives that have no limit in the EU which can be used for animal health in Australia and New Zealand. The table includes OC, once widely used, but is no longer registered for use by the statutory authorities in Australia and New Zealand^47^.

**Table 2:** EU Eco-label criteria for pesticide concentrations on greasy wool as of June 2014.

Active	Applicable EU-Ecolabel limit (ppm)
Total OC	0.5
Total SP	0.5
Total OP	2.0
Total IGR_3_	2.0
CYR	No limit
NEO	No limit
MLI	No limit
SPI	No limit

### Ectoparasiticide residue distribution parameters

**Figure 1:**
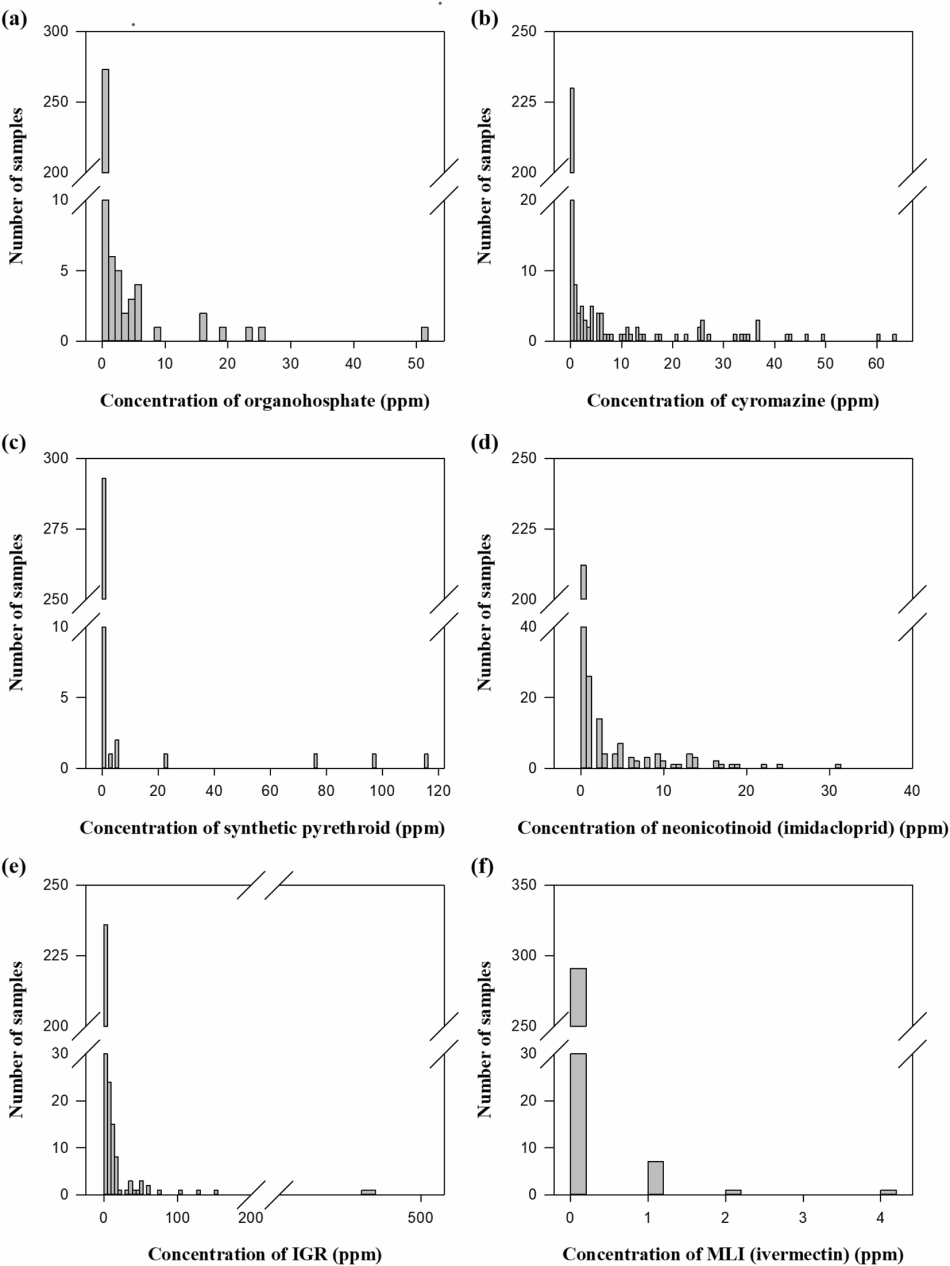
Ectoparasiticide residue distributions (**a**) OP, (**b**) CYR, (**c**) SP, (**d**) NEO, (**e**) IGR_3_, (**f**) MLI.

The distributions corresponding to each of the residue actives are shown in Fig. 1a–f, for the 2016–2017 Australian season with the corresponding descriptive statistics summarised in Table 3.

**Table 3:** Summary statistics for wool residue distribution, Australia, 2016–2017.

Active	Residue concentration values (ppm) (n = 300)								
	Min	Max	Percentiles				Mean	Skewness	Kurtosis
			Median	75th	90th	95th			
OP	0	51.8	0	0.01	0.07	3.21	0.81	8.8	94.1
SP	0	116.2	0	0	0	0	1.10	10.0	103.3
IGR_3_	0	484.0	0.22	0.46	1.56	32.92	6.95	11.9	168.9
CYR	0	63.8	0.05	0.07	0.63	25.87	3.47	3.7	14.1
NEO	0	64.0	0	0.06	0.50	13.00	2.05	5.7	45.1
MLI	0	4.0	0	0	0	0	0.04	9.7	112.9

The results show residue concentration distributions for each active are strongly asymmetrical, with a high frequency of samples where residue levels are below or close to the limit of reporting, and a relatively small proportion of samples of high residue concentration level. For each active, the mean residue concentration level is much greater than the median value and exceeds the 90th percentile of observations. The values of skewness are large and positive and the high values for kurtosis indicate the tail outliers are extremely large values compared to the mean. Collectively, these distribution shape parameters indicate the data are not normal and the mean and standard deviation values for residue concentration will not be reliable or representative of typical residue values.

### Trend analysis

**Figure 2:**
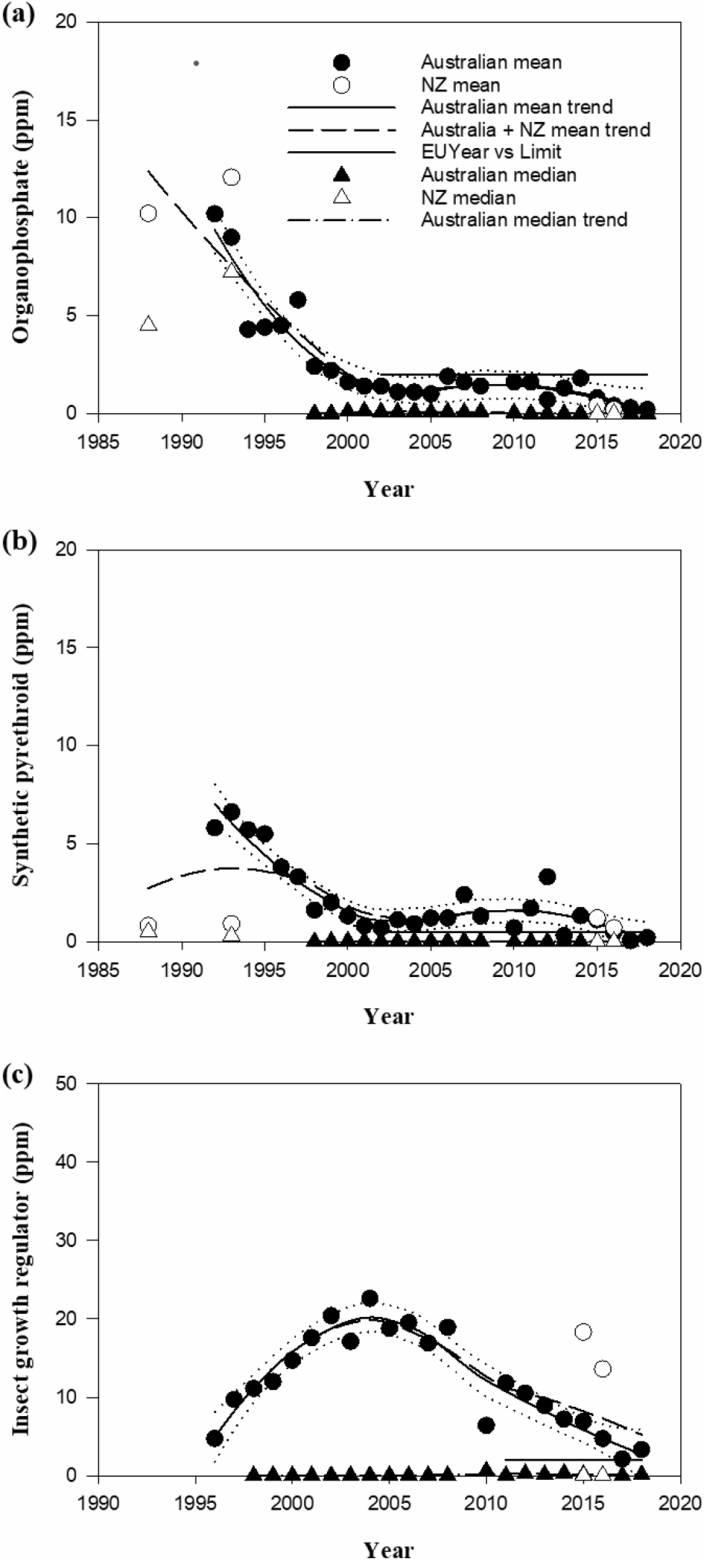
Time domain trends for greasy wool ectoparasiticide residue levels with EU limits (**a**) OP, (**b**) SP, (**c**) IGR_3_.

**Figure 3:**
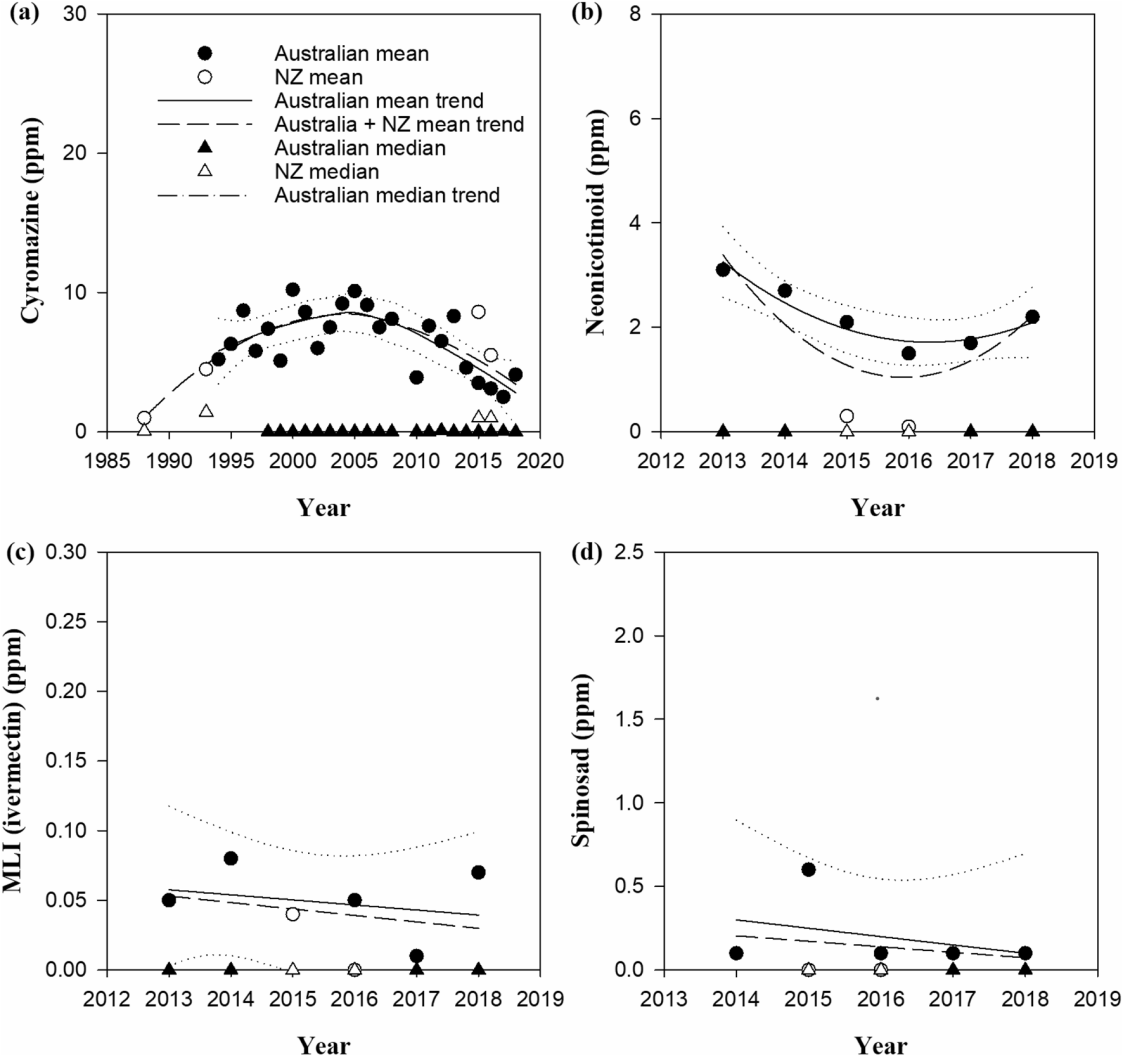
Time domain trends for greasy wool ectoparasiticide residues with no EU limits, (**a**) CYR, (**b**) NEO, (**c**) MLI, (**d**) SPI.

Australian and New Zealand data are shown in Figs. 2 and 3 with trend lines plotted according to the legend associated with each graph. The Lowess method used in the smoothing regression provides Australian trend lines (solid back line) of mean residue level of ectoparasiticides, and the 95% confidence limits of the trends (dotted black lines) to indicate the level of uncertainty associated with mean residue predictions^48^. The combined Australian and New Zealand mean trend lines (large dashed black lines) are shown on the same graphs. Median residues for Australian (black solid triangles) and New Zealand (black open triangles) data are shown with the dash/small dot trend line close to zero concentration levels.

Figures 2a–c and 3a show that mean residue levels for all actives monitored over 10 years have been trending downwards, and in particular those of OP, SP, and IGR_3_ are trending toward or within EU-Ecolabel limits. The trend median values are significantly below EU-Ecolabel residue limits for the OP, SP, IGR_3_ residues. Newer ectoparasiticides such as NEO, MLI and SPI have been monitored over 7 to 8 years and show low mean and median levels (Fig. 3b–d).

Table 4 summarises the results for the locally weighted scatterplot smoothing regression models of mean and median residue concentration trends, including goodness of fit statistics. NEO is included in Table 4 as it is representative of the newer ectoparasiticides with fewer data points at the time of publication.

**Table 4:** Regression model summary statistics for the mean and median residue levels.

Active	Mean residue concentration				Median concentration		EU-Ecolabel limit
	Australia		Australian + NZ		Australian		
	Pseudo R^2^ (%)	RSE (ppm)	Pseudo R^2^ (%)	RSE (ppm)	Pseudo R^2^ (%)	RSE (ppm)	Mean (ppm)
OP	90.4	0.852	86.0	1.336	76.2	0.027	0.5
SP	87.6	0.725	56.7	1.287	86.1	0.005	0.5
IGR_3_	90.9	2.069	70.5	2.614	86.8	0.059	2.0
CYR	73.6	1.462	57.1	1.700	85.8	0.012	No limit
NEO	90.9	0.234	70.5	0.684	100	0	No limit

The Lowess regression fit data summarised in Tables 4 and 5 indicate the method achieves relatively close fits for the mean and median data sets, but the RSE values for the regressions based on the mean values are much higher than those achieved for the median values. The RSE data are consistent with the highly skewed distributions and confirm that the mean when examined alongside the median values represent the downwards trend in residue values shown in Fig. 2, particularly over the last decade.

**Table 5:** Regression model summary for the mean predicted proportion of wool samples meeting EU-Ecolabel compliance.

Active	Australian trend		Australian + NZ trend	
	Pseudo R^2^ (%)	RSE (ppm)	Pseudo R^2^ (%)	RSE (ppm)
OP	83.4	3.175	84.9	2.966
SP	79.0	1.452	71.7	1.817
IGR_3_	90.3	4.455	87.5	4.802

### Compliance with EU residue limits

**Figure 4:**
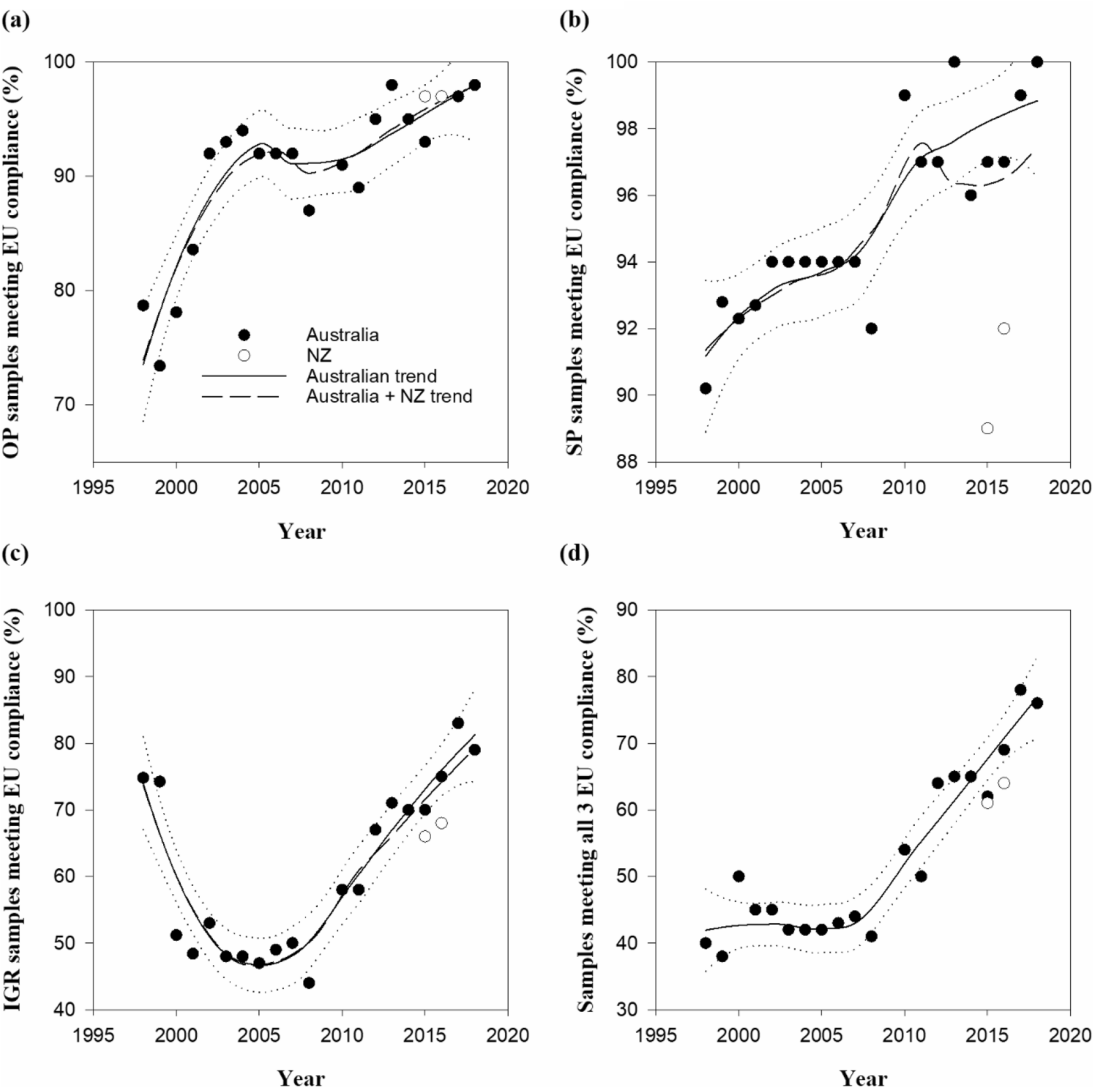
Percentage of wool production meeting EU compliance (**a**) OP, (**b**) SP, (**c**) (IGR_3_) (**d**) all actives (OP + SP + IGR_3_).

The high rates of compliance for samples meeting EU residue limits shown in Fig. 4a,b are evident from each country for OP and SP. It is clear there are lower rates of compliance corresponding to IGR regulated by the EU (Fig. 4c) with Australian compliance close to 79% (2019) and New Zealand at 68% (2017). Based on the analyses over 10 years prior to 2019 the average annual percentage increase of EU compliant wool (wool meeting EU eco label criteria for all regulated actives, OP, SP and IGR_3_ (3 actives)) from Australia was 3.2% (32% over 10 years) and the corresponding percentage when New Zealand data is included was 3.0% (30% over 10 years).

EU compliance for mean residue levels in Australian greasy farm lots for all OP, SP and IGR actives would be close to 100% in 7.5 years from 2019 and for New Zealand farm lots the expected timeline is 12 years based on the trends for the last decade. In Fig. 4d the three established IGR actives are a factor influencing the availability of compliant wool^22,49^. Until 2007/08 season IGR_2_ criterion was based on the sum of diflubenzuron + triflumuron. From 2008/09 season dicyclanil was added so the criterion increased to the sum of three insect growth regulators (IGR_3_). However alternative actives, such as SPI, are replacing the IGRs, which is reflected in the upward trends for SPI in both countries. This is also the case for the NEO active which has very low median and mean levels that are trending below 2 ppm in 2016–2017 and 2017–2018 seasons, and marginally above (2.2 ppm) in the latest 2018–2019 season.

## Discussion

The OP ectoparasiticide residues for both Australia and New Zealand greasy wool met the EU regulation maximum of 2 ppm over four recent harvesting seasons (2015/16, 2016/17, 2017/18, 2018/2019). Australian data, sampled over a longer period, has been EU compliant since changes were made to the regulations in 2002.

Median SP residues for each of the countries are below the EU limit of 0.5 ppm and the corresponding mean data in Australia for the 2018/2019 season was marginally lower than the limit while the last measured New Zealand mean result in 2016/2017 was marginally higher. The downwards trend in SP residues in Australian and New Zealand greasy fleece wool over the last 25 years has undoubtedly been influenced by deregistration of products in the SP category of actives used in sheep treatments combined with a shift to other actives regarded as more effective, such as IGR products. For example, permethrin is not registered in APVMA approved products^6^.

The means of the sum of three IGR_3_ (EU requirement) including dicyclanil, diflubenzuron and triflumuron are above the EU limit, however the median for both countries is lower than the EU limit. Dicyclanil has the higher use of these three IGR products in Australia based on APVMA registration data. In New Zealand, ACVM registered products indicate a larger use of diflubenzuron when considering the regulated IGRs. However, these actives not popular in either of the two countries.

Residues of CYR, an IGR active which is not regulated in the EU, are present at levels below the sum of the mean residue levels of dicyclanil, diflubenzuron and triflumuron. The medians for CYR, which is a popular choice of IGRs in both countries, are also low. The residues of relatively new actives, including NEO, MLI and SPI, exhibit low mean values for both countries alongside low median results. The MLI chemical, ivermectin is available in both Australia and New Zealand but usage is not as high as CYR^39,40^. SPI is also becoming established as a safe relatively new ectoparasiticide for sheep welfare.

Updated trend information utilised by the industry is expected to drive further increases in the use of alternative actives which exhibit lower residue levels and are considered by the EU to be safer for the environment. New data are historically utilised by producers, processors and manufacturers for training and extension activities leading to greater levels of availability of wool fibre meeting limits for compliance schemes administered by both governments and NGOs. New evidence of wool industry practices leading to cleaner production systems with regard to environmental criteria are available to organisations, such as MADE-BY^41^, to update environmental credentials for fibre and fabric.

## Conclusions

Animal and environmental health, alongside human health, are criteria assessed by the Australian Pesticides and Veterinary Medicines Authority and the New Zealand Ministry for Primary Industries to administer regulatory approval of agricultural compounds and veterinary medicines. Maintaining healthy sheep involves eradicating ectoparasites from the animals. Industry monitoring of ectoparasiticide residues in wool is used to measure the impact of both government and wool industry policies and practices by examining long term residue trends and determining the level of compliance with EU pesticide residue limits. Data analysed from Australia and New Zealand over the last three decades provides evidence that the practice of pest control for sheep has significantly improved through a shift away from older ectoparasiticide technology. Close to 100% of greasy wool harvested from Australia and New Zealand complies with the EU Eco-label limits for ectoparasiticide residues.

NGO organisations involved in benchmarking the environmental credentials of different types of textile fibres reference data and modelling techniques to establish indices which ultimately provide an environmental classification. Independent scientific verification of the data used for such studies provides transparency to consumers. Contemporary wool residue data taken systematically over the last three decades in Australia and New Zealand augments international procedures and regulations which have increased the supply of ‘eco-wool’ compliant fibre. The ectoparasiticide approvals processes in Australia and New Zealand examine human, animal and environmental risks and cover physico-chemical properties, toxicology, phytotoxicity, hazard assessment, product labelling and withholding periods required between animal treatment and shearing.

A standard relating to pesticide residue testing, managed by the International Wool Textile Organisation is available to participating signatory countries for monitoring residues to mitigate and eliminate environmental and human health impacts from the use of animal health treatments. The acquisition of relevant data for modelling and classification of fibre types adds to a credible resource that can be analysed periodically to assess progress with wool residue management. The Australian and New Zealand wool residue monitoring data updates information showing positive downward trends and evidence of cleaner practices.

## Data Availability

The datasets used and/or analysed during the current study are available from the corresponding author on reasonable request.
